# Fasting glucose mediates the influence of genetic variants of *SOD2* gene on lean non-alcoholic fatty liver disease

**DOI:** 10.3389/fgene.2022.970854

**Published:** 2022-10-18

**Authors:** Na Wu, Xiangyu Zhai, Fan Yuan, Jie Li, Ning Yu, Fengwei Zhang, Dong Li, Jianying Wang, Lei Zhang, Yi Shi, Guang Ji, Guang He, Baocheng Liu

**Affiliations:** ^1^ Shanghai Innovation Center of Traditional Chinese Medicine Health Service, Shanghai University of Traditional Chinese Medicine, Shanghai, China; ^2^ Bio-X Institutes, Key Laboratory for the Genetics of Developmental and Neuropsychiatric Disorders, Shanghai Jiao Tong University, Shanghai, China; ^3^ Zhangjiang Community Health Service Center of Pudong New District, Shanghai, China; ^4^ Institute of Digestive Diseases, Longhua Hospital, Shanghai University of Traditional Chinese Medicine, Shanghai, China; ^5^ Graduate School of Sport Sciences, Waseda University, Saitama, Japan

**Keywords:** *SOD2* gene, genetic variants, fasting glucose, mediation effect, lean non-alcoholic fatty liver disease

## Abstract

**Background:** Non-alcoholic fatty liver disease (NAFLD) imposes an enormous burden on public health, and a large proportion of NAFLD patients are lean with normal body weight, which is rarely mentioned. We conducted this study to determine the mediation effects of fasting glucose on the relationships between genetic variants of *SOD2* and the susceptibility of lean NAFLD in the elderly Chinese Han population.

**Methods:** Data in this manuscript were collected in a cross-sectional study among 5,387 residents (aged ≥60 years) in the Zhangjiang community center, Shanghai, China, in 2017. Ten (single nucleotide polymorphisms) SNPs previously reported to be related to NAFLD and obesity, including rs9939609, rs1421085, rs9930506, rs626283, rs641738, rs4880, rs58542926, rs738409, rs2281135, and rs2294918 were genotyped. The associations between genetic variations in *SOD2* and fasting glucose in five genetic models were analyzed with the SNPassoc R package and rechecked with regression analysis. Mediation models were conducted to explore whether fasting glucose can mediate the association between SNPs and the susceptibility of lean NAFLD.

**Results:** In this study, lean NAFLD individuals had a higher waist circumference and waist-to-hip ratio, ALT, and fasting glucose than lean non-NAFLD individuals (*p* < 0.050). In comparison, the AA genotypic frequency of rs4880 in *SOD2* gene was much lower in lean NAFLD patients (*p* = 0.005). And rs4800 had a significant indirect effect on lean NAFLD incidence mediated by fasting glucose (*p* < 0.001).

**Conclusion:** For the first time, the mediation effect of fasting glucose on the association of rs4880 in *SOD2* with the susceptibility of lean NAFLD was clarified in the elderly Chinese Han population. It emphasized the connection between glucose homeostasis and oxidative stress in the mechanisms of lean NAFLD.

## Introduction

Non-alcoholic fatty liver disease (NAFLD) imposes an enormous burden on public health. Evidence shows that NAFLD is triggered by combined genetic and epigenetic changes induced by environmental factors (Eslam et al., 2018). Notably, quite a number of NAFLD patients are lean with normal body weight (Kim et al., 2004).

The prevalence of lean NAFLD varies in different regions. In a retrospective study from the United States including 11,613 participants, the prevalence of NAFLD among non-lean subjects was 28.8%, whereas it was 9.67% in the lean subjects (Younossi et al., 2012). A study from rural Italy showed that 75% of NAFLD patients had a body mass index (BMI) below 25 kg/m^2^ (Das et al., 2010). In Asia, the prevalence of non-obese NAFLD and lean NAFLD was 12.6% (Kwon et al., 2012) and 16% (Sinn et al., 2012) (BMI <25 kg/m^2^ and <23 kg/m^2^, respectively) in Korea; the prevalence of non-obese NAFLD was 12.6% in Japan; among the lean participants, 33.7% had NAFLD in India (Choudhary et al., 2021); 15.2% NAFLD patients were non-obese in Japan (Nishioji et al., 2015); the prevalence of non-obese NAFLD was 19.4% in Hong Kong (Wei et al., 2015) and 11.7% in the mainland (Wu et al., 2020) of China. Notably, lean NAFLD was more prevalent in non-alcoholic steatohepatitis (NASH), ranging from 43 to 53% (Wang et al., 2019). The heterogeneity of prevalence in lean or non-obese NAFLD seems to be an outcome of diagnostic strategy selection and genetic variation induced by ethnicity (Wang et al., 2019; Ye et al., 2020; Vilarinho et al., 2021).

The pathogenesis of NAFLD in the lean subtype is likely driven by the accumulation of free fatty acids, which result from excessive caloric intake and accelerated adipose lipolysis and lead to insulin resistance and expanded visceral adiposity (Vilarinho et al., 2021). Beyond that, genetic polymorphisms also influence the manifestation of lean NAFLD (Vilarinho et al., 2021). The prevalence of rs738409 in patatin-like phospholipase domain-containing 3 (*PNPLA3*) gene was much higher in lean NAFLD patients than in obese NAFLD or non-obese controls (Zou et al., 2020). The transmembrane 6 superfamily 2 (*TM6SF2*) rs58542926 was reported to have a direct relationship with lean NAFLD (Chen et al., 2020a); further, this variation is also associated with lower body mass index (BMI) (Chen et al., 2020b). In addition, variants in membrane bound O-acyltransferase domain containing 7 (*MBOAT7*), fat mass and obesity-associated (*FTO*), and superoxide dismutase 2 (*SOD2*) which have been reported to be involved in the regulation of inflammatory lipid pathways, lipogenesis, and oxidative stress, respectively, contributed to variation in NAFLD, with the most severe being non-alcoholic steatohepatitis (NASH) and fibrosis (Al-Serri et al., 2012; Guo et al., 2013; Meroni et al., 2020; Teo et al., 2021). However, no data or replicated research on the function of the above genetic variation in candidate genes in lean NAFLD patients have been conducted.

There is convincing evidence that elevated glucose level and dyslipidemia that accompany the disease development are tightly involved in the pathogenesis of NAFLD at almost every step of the steatotic and inflammatory process (Rosso et al., 2019). Chronic inflammation is currently considered as one of the key factors in NAFLD development and is present starting from the earliest stages of the pathology initiation. It may also be regarded as one of the possible links between NAFLD and type II diabetes (T2D) (Stefan and Cusi, 2022). Against this background, we undertook this study to further elucidate the complex interplay between impaired glucose regulation (e.g., fasting glucose), inflammation (e.g., *SOD2*) and hepatic damage in a well-characterized cohort of NAFLD patients.

To further examine this biologically plausible association between above-mentioned polymorphism in candidate genes and susceptibility to lean NAFLD, we performed a classical case-control association study in unrelated patients with lean NAFLD in the elderly Chinese Han population.

## Materials and methods

### Study participants

Data were collected in a cross-sectional study among 5,387 residents (aged ≥60 years) in the Zhangjiang community center, Shanghai, China, in 2017. The study was approved by the Ethics Committee of Shanghai University of Traditional Chinese Medicine. Written informed consent was provided before the examination.

The local residents in Shanghai who can complete the data measurements were included in this study. And participants with mental disorders, malignant tumors, or incomplete recorded information would be excluded. After an initial screening, 5,338 potential subjects were included. Then, 1,449 participants were randomly chosen for the genotyping analysis. However, 230 participants lacked BMI data, abused alcohol (≥140 g/week in males and ≥70 g/week in females), were carriers of hepatitis B or C, or had a history of drug-induced liver disease or autoimmune liver disease were excluded. Ultimately, 1,219 participants (NAFLD, *n* = 750; non-NAFLD, *n* = 469) were included in the final analysis. According to the classification of adult Asian populations (Fan et al., 2017), the lean individuals in this study were defined by body mass index (BMI) < 23 kg/m^2^. Then, participants were categorized into four groups: lean NAFLD (BMI <23 kg/m^2^, *n* = 106), non-lean NAFLD (BMI ≥23 kg/m^2^, *n* = 644), lean non-NAFLD (BMI <23 kg/m^2^, *n* = 216) and non-lean non-NAFLD (BMI ≥23 kg/m^2^, *n* = 253). Finally, lean NAFLD and lean non-NAFLD individuals were included for further analysis.

### Measurement

Color ultrasound system (Philips IU22) was used for the diagnosis of NAFLD, the probe frequency was set as 3.5–5.5 MHz. All the patients were in the supine position, and the liver was routinely scanned in multiple slices to observe the liver size, echo of liver parenchyma and far-field. As normal liver parenchyma is the same as or slightly more echogenic (“brighter”) than the adjacent kidney and spleen (Zwiebel, 1995), ultrasound beam scattering by lipid droplets in steatosis causes more echo signals to return to the transducer, creating the appearance of a “bright” or hyperechoic liver (Charatcharoenwitthaya and Lindor, 2007). Additionally, fat also attenuates the beam which decreases beam penetration into tissue, and this attenuation leads to poor visualization of structures within the steatotic liver parenchyma (e.g., intrahepatic vessels and bile ducts) and structures deep to the liver (e.g., diaphragm). Thus, the presence of steatosis can be inferred if the liver is too bright and/or if liver structures are blurry or poorly visualized. As a common examination method, color ultrasound system has the advantages of safety and non-invasiveness, and it has a high patient tolerance and will not damage the patient’s health. The cost of color ultrasound examination is low and can be repeated without increasing the financial burden of patients, making it widely used in clinical diagnosis and treatment. According to the guidelines for the diagnosis and treatment of nonalcoholic fatty liver disease, the diagnostic criteria for color ultrasound are as follows: 1) Diffuse echo will be enhanced near the liver of the patient, which is stronger than that of the kidney tissue; 2) The structure of the intrahepatic duct cannot be clearly observed; 3) The echo of far-field is attenuated. If two of the above three items are satisfied, the diagnosis can be made (Fan et al., 2011).

Information, i.e., age, gender, alcohol consumption, smoking, and medical history was acquired by questionnaire. BMI was calculated as weight (kg) divided by height squared (m^2^). A non-stretch tape was used to measure waist and hip circumference by the trained professional. Electronic sphygmomanometers (Biospace, Cheonan, South Korea) were used to measure the diastolic and systolic blood pressure (DBP and SBP). Fasting glucose (Hexokinase activity assay kit, ab136957), alanine transaminase (ALT), aspartate transaminase (AST), total cholesterol (TC), low-density lipoprotein (LDL), high-density lipoprotein (HDL) and triglyceride (TG) were measured using the biochemistry analyzer (Hitachi, Tokyo, Japan). Reagents for glucose, ALT, AST, TC, LDL, HDL, and TG detection were from Wako Pure Chemical Corporation, Japan. The quality control materials were provided by Beckman Company (M507471 and M507473), and the calibrators were provided by Wako Pure Chemical Corporation, Japan.

### Genotyping

Genomic DNA was extracted from venous blood leukocytes using the EZ1 DNA Blood 350 μl kit (Qiagen) according to the instructions. Ten (single nucleotide polymorphisms) SNPs including: rs9939609, rs1421085 and rs9930506 in *FTO*, rs626283 and rs641738 in *MBOAT7*, rs4880 in *SOD2*, rs58542926 in *TM6SF2* and rs738409, rs2281135 and rs2294918 in *PNPLA3* from NCBI database of SNP database (www.ncbi.nlm.nih.gov/SNP) were analyzed. And all these above genes were reported to be strongly link to NAFLD or obesity traits (Anstee et al., 2020; Loos and Yeo, 2022). Matrix-assisted laser desorption/ionization time-off light mass spectrometer in MassARRAY Analyzer four platforms (Sequenom, San Diego, CA) was used for genotyping. Probes and primers were designed with online Assay Design Suite version 2.0 software. The polymerase chain reaction was performed according to the instructions of the manufacturers. More detailed information about primers and polymerase chain reaction conditions is available upon request.

### Statistical analysis

Participants’ basic traits were presented with mean, standard deviation (SD), and confidence interval (CI). Independent samples *t*-test was adopted for the group comparison. Categorical data were calculated as a percentage. Non-normally distributed data were analyzed by converting log to normally distributed data, and non-parametric testing, i.e., Mann-Whitney U test, was used for data with non-normal distributions. Allelic and genotypic distributions and Hardy-Weinberg equilibrium were analyzed with the online software SHEsis (http://analysis.bio-x.cn/myAnalysis.php) (Shi and He, 2005), and all the above SNPs met Hardy-Weinberg equilibrium (*p* > 0.050).

“SNPassoc” R package (version 2.0–11) was applied for the association analysis between SNPs with phenotypes in five genetic models (codominant, dominant, recessive, over-dominant and log-additive models, respectively) (Gonzalez et al., 2007). The logistic regression analysis was used to verify the association of fasting glucose with rs4880 of *SOD2* in lean NAFLD. Only those variables that are statistically significant in both genetic association and regression analysis will be included in the subsequent mediation analysis. Mediation models conducted with mediation package (version 4.5.0) in R software (version 3.6.3) (Supplementary Table S1) were used to explore whether specific phenotypes can mediate the association between SNPs and the susceptibility of lean NAFLD after adjusting gender. *p* < 0.05 was considered significant in this study. We applied a Benjamini–Hochberg false discovery rates (FDR) correction to correct for multiple comparisons. *p* value less than 0.05 but did not survive the FDR correction was considered to be suggestive of a potential association.

## Results

### Participant characteristics

322 lean individuals were included in this sub-analysis (Table 1). The average age of lean NAFLD (*n* = 106) and lean non-NAFLD (*n* = 216) individuals was 72.5 and 73.5 years old. Anthropometric traits such as weight, BMI, waist circumference, hip circumference and waist to hip ratio (*p* < 0.001) and serum traits such as ALT, fasting glucose, hemoglobin, TC, LDL, and TG (*p* < 0.050) were significantly lower in lean non-NAFLD individuals than lean NAFLD individuals. While lean non-NAFLD individuals had increased HDL (*p* < 0.001). While there were no significant differences in blood pressure, AST and the percentage of hypertension, T2D and hyperlipidemia between lean NAFLD and lean non-NAFLD individuals.

**TABLE 1:** Characteristics of lean NAFLD and non-NAFLD individuals.

Individuals characteristics	Lean NAFLD		Lean non-NAFLD		Mean difference [95% CI]
	Mean or N (%)	SD	Mean or N (%)	SD	
N	106		216		
Age (Year)	72.54	6.05	73.54	6.15	−0.999 [−2.427, 0.428]
Gender %					
Female	64.20%		58.80%		
Male	35.80%		41.20%		
Height (cm)	159.13	8.24	158.60	8.35	0.535 [−1.405, 2.475]
Weight (kg)	55.42	6.25	51.92	6.98	3.497 [1.923, 5.070]**
BMI (kg/m^2^)	21.83	0.96	20.60	1.78	1.232 [0.932, 1.533]**
Waist circumference (cm)	79.94	6.26	75.63	6.92	4.318 [2.740, 5.896]**
Hip circumference (cm)	90.43	3.91	88.37	4.85	2.062 [1.064, 3.058]**
Waist-to-hip ratio	0.88	0.06	0.85	0.08	0.033 [0.016, 0.049]**
SBP (mmHg)	142.04	20.43	138.04	22.45	4.001 [−1.108, 9.110]
DBP (mmHg)	81.08	12.32	78.83	11.92	2.244 [−0.579, 5.066]
HR	75.45	7.51	76.12	8.05	−0.677 [−2.652, 1.298]
ALT (U/L)	22.44	9.25	19.73	9.28	2.712 [0.548, 4.876]*
AST (U/L)	23.88	10.44	23.53	8.58	0.350 [−1.804, 2.503]
Fasting glucose (mmol/L)	6.70	2.20	5.89	1.66	0.813 [0.336, 1.290]*
Hemoglobin (g/L)	139.08	13.69	134.00	13.45	5.075 [1.919, 8.232]*
TC (mmol/L)	5.29	0.91	4.99	1.01	0.298 [0.078, 0.518]*
HDL (mmol/L)	1.16	0.20	1.33	0.30	−0.171 [−0.227, -0.116]**
LDL (mmol/L)	3.30	0.85	3.05	0.89	0.248 [0.043, 0.452]*
TG (mmol/L)	1.92	1.20	1.18	0.69	0.751 [0.503, 0.100]**
Hypertension%	55.70		44.00		
T2D%	20.80		12.00		
Hyperlipidemia%	10.40		6.50		

*p* values are based on independent sample *t*-test. ALT, alanine aminotransferase; AST, aspartate aminotransferase; BMI, body mass index; DBP, diastolic blood pressure; HDL, high density lipoprotein; LDL, low density lipoprotein; NAFLD, nonalcoholic fatty liver disease; SBP, systolic blood pressure; TC, total cholesterol; TG, triglyceride; T2D, type 2 diabetes. * and ** indicate *p* value <0.050 and 0.001, respectively.

### Genetic association between SNPs and lean non-alcoholic fatty liver disease

Ten tested SNPs i.e., rs9939609, rs1421085, rs9930506, rs626283, rs641738, rs4880, rs58542926, rs738409, rs2281135, and rs2294918 are shown in Table 2. The allele and genotype distributions of these SNPs are presented in Table 3. Although there were no significant differences in the frequency of A allele and G allele of rs4880 in *SOD2* gene between lean NAFLD and lean non-NAFLD individuals, the AA genotypic frequency of rs4880 in SOD2 gene was much lower in lean NAFLD patients than lean non-NAFLD individuals (*p* = 0.005, FDR adjusted *p* = 0.059). And the other SNPs’ allele and genotype frequencies, i.e., rs9939609, rs1421085 and rs9930506 in *FTO*, rs626283 and rs641738 in *MBOAT7*, rs58542926 in *TM6SF2*, and rs738409, rs2281135 and rs2294918 in *PNPLA3* were no significant differences between lean NAFLD patients and lean non-NAFLD individuals.

**TABLE 2:** The twelve SNPs analyzed in this study.

Gene	SNP ID	Chromosome	Function	Allele
FTO	rs9939609	Chr16:53786615	intron_variant	T/A
	rs1421085	Chr16:53767042	intron_variant	T/C
	rs9930506	Chr16:53796553	intron_variant	A/G
MBOAT7	rs626283	Chr19:54173307	intron_variant	G/C
	rs641738	Chr19:54173068	intron_variant	C/T
SOD2	rs4880	Chr6:159692840	missense_variant	A/G
TM6SF2	rs58542926	Chr19:19268740	missense_variant	C/T
PNPLA3	rs738409	Chr22:43928847	missense_variant	G/C
	rs2281135	Chr22:43936690	intron_variant	A/G
	rs2294918	Chr22:43946236	missense_variant	G/A

FTO, Fat mass and obesity associated; MBOAT7, Membrane bound O-acyltransferase domain containing 7; PNPLA3, Patatin like phospholipase domain containing 3; SNP, Single nucleotide polymorphisms; SOD2, Superoxide dismutase 2; TM6SF2, Transmembrane 6 superfamily member 2.

**TABLE 3:** Analyzed genes allele and genotype distribution in lean individuals.

Gene	SNP	Allele frequency		χ2	P	FDR adjusted	OR [95% CI]	Genotype frequency			χ2	P	FDR adjusted
FTO	rs9939609	T	A	2.209	0.137	0.401	1.463 [0.883, 2.425]	T/T	A/T	A/A	2.774	0.249	0.499
	Lean NALFD	183 (0.863)	29 (0.136)					79 (0.745)	25 (0.235)	2 (0.018)			
	Lean non-NAFLD	388 (0.902)	42 (0.097)					174 (0.809)	40 (0.186)	1 (0.004)			
FTO	rs1421085	T	C	0.659	0.416	0.787	1.243 [0.734, 2.103]	T/T	T/C	C/C	1.68	0.431	0.863
	Lean NALFD	181 (0.878)	25 (0.121)					80 (0.776)	21 (0.203)	2 (0.019)			
	Lean non-NAFLD	378 (0.900)	42 (0.100)					169 (0.804)	40 (0.190)	1 (0.004)			
FTO	rs9930506	A	G	1.189	0.275	0.546	1.259 [0.831, 1.909]	A/A	A/G	G/G	1.162	0.559	0.738
	Lean NALFD	168 (0.792)	44 (0.207)					67 (0.632)	34 (0.320)	5 (0.047)			
	Lean non-NAFLD	356 (0.827)	74 (0.172)					148 (0.688)	60 (0.279)	7 (0.032)			
SOD2	rs4880	A	G	0.420	0.516	0.949	1.172 [0.724, 1.897]	A/A	A/G	G/G	10.265	0.005	0.059
	Lean NALFD	182 (0.858)	30 (0.141)					83 (0.783)	16 (0.150)	7 (0.066)			
	Lean non-NAFLD	377 (0.876)	53 (0.123)					164 (0.762)	49 (0.227)	2 (0.009)			
TM6SF2	rs58542926	C	T	0.001	0.967	0.970	1.014 [0.497, 2.071]	C/C	C/T		0.001	0.966	0.968
	Lean NALFD	200 (0.943)	12 (0.056)					94 (0.886)	12 (0.113)				
	Lean non-NAFLD	406 (0.944)	24 (0.055)					191 (0.888)	24 (0.111)				
MBOAT7	rs626283	G	C	0.547	0.459	0.723	1.151 [0.792, 1.671]	G/C	G/G	C/C	1.161	0.559	0.812
	Lean NALFD	154 (0.726)	58 (0.273)					44 (0.415)	55 (0.518)	7 (0.066)			
	Lean non-NAFLD	324 (0.753)	106 (0.246)					76 (0.353)	124 (0.576)	15 (0.069)			
MBOAT7	rs641738	C	T	0.603	0.437	0.949	1.160 [0.796, 1.690]	C/T	T/T	C/C	1.245	0.536	0.908
	Lean NALFD	153 (0.728)	57 (0.271)					43 (0.409)	7 (0.066)	55 (0.523)			
	Lean non-NAFLD	324 (0.757)	104 (0.242)					74 (0.345)	15 (0.07)	125 (0.584)			
PNPLA3	rs738409	G	C	0.027	0.868	0.949	1.029 [0.729, 1.453]	G/G	C/G	C/C	3.421	0.180	0.695
	Lean NALFD	75 (0.357)	135 (0.642)					16 (0.152)	43 (0.409)	46 (0.438)			
	Lean non-NAFLD	150 (0.35)	278 (0.649)					21 (0.098)	108 (0.504)	85 (0.397)			
PNPLA3	rs2281135	A	G	0.165	0.683	0.949	0.931 [0.661, 1.311]	A/A	A/G	G/G	1.25	0.535	0.908
	Lean NALFD	76 (0.361)	134 (0.638)					15 (0.142)	46 (0.438)	44 (0.419)			
	Lean non-NAFLD	162 (0.378)	266 (0.621)					27 (0.126)	108 (0.504)	79 (0.369)			
PNPLA3	rs2294918	G	A	0.093	0.759	0.949	0.935 [0.610, 1.432]	G/G	A/G	A/A	0.183	0.912	0.968
	Lean NALFD	174 (0.820)	38 (0.179)					71 (0.669)	32 (0.301)	3 (0.028)			
	Lean non-NAFLD	347 (0.810)	81 (0.189)					141 (0.658)	65 (0.303)	8 (0.037)			

FDR, False discovery rate; FTO, Fat mass and obesity associated; MBOAT7, Membrane bound O-acyltransferase domain containing 7; PNPLA3, Patatin like phospholipase domain containing 3; SNP, Single nucleotide polymorphisms; SOD2, Superoxide dismutase 2; TM6SF2, Transmembrane 6 superfamily member 2.

### Association of SNPs with phenotypes in lean non-alcoholic fatty liver disease individuals

The association using five genetic models is presented in Table 4. Only *SOD2* polymorphism rs4880 was significantly associated with fasting glucose under the dominant and over-dominant models. The AA and AA-GG genotypes of *SOD2* rs4880 polymorphism were statistically related to higher fasting glucose (*p* = 0.029 and *p* = 0.024, respectively). Information about associations between rs4880 and other phenotypes is shown in Supplementary Table S2. And there was no significant relation between rs4880 and phenotypes (i.e., hemoglobin, ALT, HDL, LDL, and TG).

**TABLE 4:** Association between rs4880 in *SOD2* and fasting glucose.

Genotype	N	Mean difference [95%CI]	P
Codominant			
A/A	245		0.077
A/G	63	−0.608 [−1.133, −0.084]	
G/G	8	−0.167 [−1.501, 1.167]	
Dominant			
A/A	245		0.029
A/G-G/G	71	−0.558 [−1.059, −0.058]	
Recessive			
A/A-A/G	308		0.950
G/G	8	−0.043 [−1.381, 1.296]	
Over-dominant			
A/A-G/G	253		0.024
A/G	63	−0.603 [−1.125, −0.081]	
log-Additive			
0,1,2		−0.413 [−0.841, 0.015]	0.060

CI, Confidence interval; SNP, Single nucleotide polymorphisms; SOD2, Superoxide dismutase 2.

Further, we used rs4880 as a predictor to examine the association with fasting glucose using logistic regression. And rs4880 genotype was still significantly associated with fasting glucose (*β* = 0.607, *R* ^2^ = 0.011, *p* = 0.024) after adjusting gender and age (Table 5).

**TABLE 5:** Regression analysis on the impacts of rs4880 of *SOD2* on fasting glucose.

Gene	SNP	Phenotype	Statistics of regression analyses			
			β	*R* ^2^	t	P
SOD2	rs4880	Fasting glucose	0.607	0.011	2.270	0.024

SNP, Single nucleotide polymorphisms; SOD2, Superoxide Dismutase 2.

### The possible mediation effect of fasting glucose on the association between rs4880 and the susceptibility of lean non-alcoholic fatty liver disease

**FIGURE 1:**
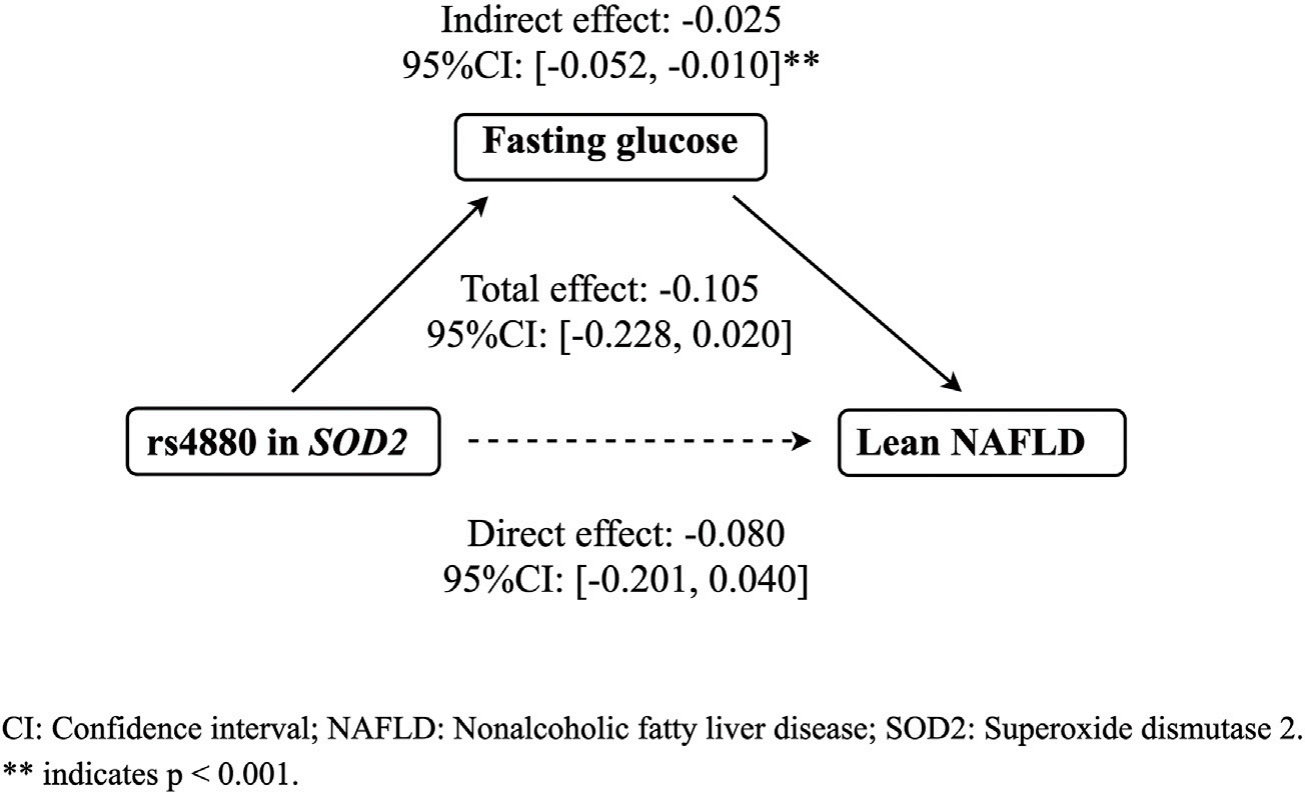
The mediation effect of fasting glucose on the association between rs4880 and the risk of lean NAFLD. CI: Confidence interval; Nonalcoholic fatty liver disease; SOD2: Superoxide dismutase 2.

The mediation analysis indicated that rs4880 had no significant direct effect on lean NAFLD (*β* = −0.080, 95%CI: [−0.201, 0.040]), while rs4800 had a significant indirect effect on lean NAFLD incidence through fasting glucose (*β* = −0.025, 95%CI: [−0.052, −0.010], *p* < 0.001) (Figure 1).

## Discussion

In this retrospective case-control study, we observed a significant difference in the genotype distribution of rs4880 in *SOD2* between lean NAFLD and lean non-NAFLD individuals. Additionally, for the first time, we explored that the association between rs4880 and the risk of lean NAFLD was mediated by fasting glucose in the elderly Chinese Han population. It may imply that fasting glucose warrants careful control, evaluation, and follow-up to prevent lean NAFLD.

Previous studies on genetic variation of *SOD2* have mainly focused on obesity (Lewandowski et al., 2020), T2D (Flekac et al., 2008), NASH (Huang et al., 2021) and even fibrosis (Al-Serri et al., 2012). \Lewandowski et al. (2020) observed that nearly 90% of obese participants were CT genotype of rs4880 in *SOD2*. Flekac et al. (2008) investigated the serum SOD activity and frequency of allele and genotype of *SOD2* between T2DM and healthy controls, and found that the SOD activity was higher in CC genotype of SOD2 gene than TT. Huang et al. (2021) reported that the NASH patients had a higher prevalence of *SOD2* C allele (38.8%) compared with simple steatosis (25.0%) and healthy controls (22.9%). While Vespasiani-Gentilucci et al. (2018) described no differences in *SOD2* rs4880 among NASH-cirrhosis, noncirrhotic NAFLD and healthy controls. No significant allelic distribution between lean NAFLD and lean non-NAFLD individuals was found in the present study. However, AA genotype of rs4880 (78.3%) in lean NAFLD patients was higher than AG or GG genotype. Our study complemented the above genetic studies with lean NAFLD samples, and elucidated the susceptibility of *SOD2* rs4880 in lean NAFLD, then emphasized the importance of inflammation in lean NAFLD as *SOD2* plays a critical role in evoking anti-oxidative stress, which has been implicated in the pathogenesis of many metabolic diseases (Fukai and Ushio-Fukai, 2011).

The influence of SNPs in *SOD2* on redox status, which may affect metabolic diseases, has emerged in recent years. Both *SOD2* gene expression and enzyme level decreased in T2DM who had higher fasting glucose than healthy controls (Suri et al., 2021). Gonzalez-Menendez et al. (2018) demonstrated that the overexpression of glucose transporter gene could maintain the activity of *SOD2* under the circumstance of lacking glucose. Instead of NAFLD, Flores et al. (2017) proposed that the polymorphism in *SOD2* may lead to dyslipidemia and hyperglycemia in stroke. After heterozygous *SOD2* deletion, Kang et al. (2014) showed that a 16-week high fat diet contributes to the impairment of glucose-stimulated insulin secretion; and Hoehn et al. (2009) reported that the chow diet impaired the glucose tolerance. In addition, upregulation of microRNA-21 caused by 1-week oscillating high glucose exposure could provoke the disrupted reactive oxygen species (ROS) homeostasis appearing as an inhibited expression of *SOD2* (La Sala et al., 2018). The present study explored the mediation effect of fasting glucose on the association between rs4880 in *SOD2* and the susceptibility of lean NAFLD. Combined with the observation, which showed a decreased expression of *SOD2* in human umbilical vein endothelial cells (HUVECs) after oscillating high glucose (La Sala et al., 2016), it suggests that pathoglycemia and antioxidant defense co-exist or interact in lean NAFLD. In our study, lean elderly NAFLD patients had higher fasting glucose than lean non-NAFLD participants, which was consistent with the previous studies. Lean middle-aged NAFLD patients were prone to develop an elevated glucose level (Aneni et al., 2020). While, lean middle-aged NAFLD patients had lower fasting blood glucose than non-lean NAFLD controls (Chen et al., 2020a). All these findings may support the consensus that poor glycemic control must be avoided to prevent NAFLD, especially the lean individuals. A better understanding of the molecular mechanisms underpinning ROS homeostasis, especially the role of polymorphisms in *SOD2,* will improve glucose control.

The first strength of our study is that NAFLD was diagnosed with a stringent color ultrasound system and not dependent on the substitute markers such as serum alanine aminotransferase. Furthermore, it is the first retrospective and community-based study to report the mediation effect of fasting glucose on the association between rs4880 in *SOD2* and the susceptibility of lean NAFLD in the elderly Chinese Han population. There are, however, several limitations. Only ten SNPs were included in this study, and more genomic polymorphisms should be considered in the future; the consideration of muscle mass loss failed to account for the elderly participants, but muscle mass loss might limit the utility of BMI as a measure of adiposity in the elderly; the genomic association study was only conducted in the elderly Chinese Han population. A larger sample size with different races and ages needs to be considered to raise the reliability of the current finding.

## Conclusion

In conclusion, for the first time, the possible mediation effect of fasting glucose on the association of rs4880 in *SOD2* with the susceptibility of lean NAFLD was clarified in the elderly Chinese Han population. It emphasized the connection between glucose homeostasis and oxidative stress in the mechanisms of lean NAFLD. Further longitudinal research should be established to examine the cause and effect in the relationship between genetic variation, glucose and the susceptibility to lean NAFLD.

## Data Availability

The datasets presented in this study can be found in online repositories. The names of the repository/repositories and accession number(s) can be found in the article/Supplementary Material.

## Supplementary material

SUPPLEMENTARY TABLE S1 R script for mediation analysis.
