# Supplementary material for: Fasting glucose mediates the influence of genetic variants of SOD2 gene on lean non-alcoholic fatty liver disease
Source: Front Genet. 2022 Oct 18;13:970854. doi: 10.3389/fgene.2022.970854 (PMC9622784; doi:10.3389/fgene.2022.970854)
Supplement: Supplementary file 1 [file Table1.DOCX]

**Supplementary Table 1**

| R package | Information |
| --- | --- |
| Mediation | dat_l_all <- dat[which(dat$BMIlevel == "lean"),]  dat_l <- as.data.frame(dat_l_all[,c(1:28,167,209)])  dat_l_t <- setupSNP(dat_l, colSNPs = 29, sep = "")  med.fit <- lm(fasting_glucose ~ rs4880 + gender + age, data = dat_l_t)  out.fit <- glm(NAFLD1 ~ rs4880 + fasting_glucose + gender + age, data = dat_l_t, family = binomial("probit"))  set.seed(2022)  med.out <- mediate(med.fit, out.fit, treat = "rs4880", mediator = "fasting_glucose",  boot = T, sims = 1000)  summary(med.out) |
