# Supplementary material for: Fasting glucose mediates the influence of genetic variants of SOD2 gene on lean non-alcoholic fatty liver disease
Source: Front Genet. 2022 Oct 18;13:970854. doi: 10.3389/fgene.2022.970854 (PMC9622784; doi:10.3389/fgene.2022.970854)
Supplement: Supplementary file 2 [file Table2.docx]

**Supplementary Table 2** Association between rs4880 of *SOD2* and other phenotypes under five genetic models in lean NAFLD individuals

| **Phenotype** | **Genotype** | **N** | **Mean difference[95%CI]** | | **P** |
| --- | --- | --- | --- | --- | --- |
| Hemoglobin | Codominant |  |  | |  |
|  | A/A | 245 |  | 0.369 | |
|  | A/G | 63 | 0.547[-3.261, 4.356] |  | |
|  | G/G | 8 | 6.938[-2.747, 16.624] |  | |
|  | Dominant |  |  |  | |
|  | A/A | 245 |  | 0.495 | |
|  | A/G-G/G | 71 | 1.268[-2.369, 4.904] |  | |
|  | Recessive |  |  |  | |
|  | A/A-A/G | 308 |  | 0.166 | |
|  | G/G | 8 | 6.826[-2.814, 16.466] |  | |
|  | Over-dominant |  |  |  | |
|  | A/A-G/G | 253 |  | 0.866 | |
|  | A/G | 63 | 0.328[-3.474, 4.130] |  | |
|  | log-Additive |  |  |  | |
|  | 0,1,2 |  | 1.635[-1.473, 4.742] | 0.303 | |
| ALT | Codominant |  |  |  | |
|  | A/A | 245 |  | 0.610 | |
|  | A/G | 63 | -1.100[-3.668, 1.468] |  | |
|  | G/G | 8 | -2.003[-8.534, 4.529] |  | |
|  | Dominant |  |  |  | |
|  | A/A | 245 |  | 0.337 | |
|  | A/G-G/G | 71 | -1.201[-3.648, 1.245] |  | |
|  | Recessive |  |  |  | |
|  | A/A-A/G | 308 |  | 0.593 | |
|  | G/G | 8 | -1.778[-8.285, 4.730] |  | |
|  | Over-dominant |  |  |  | |
|  | A/A-G/G | 253 |  | 0.428 | |
|  | A/G | 63 | -1.036[-3.594, 1.521] |  | |
|  | log-Additive |  |  |  | |
|  | 0,1,2 |  | -1.063[-3.156, 1.029] | 0.320 | |
| HDL | Codominant |  |  | 0.198 | |
|  | A/A | 245 |  |  | |
|  | A/G | 63 | 0.046[-0.033, 0.125] |  | |
|  | G/G | 8 | -0.134[-0.335, 0.067] |  | |
|  | Dominant |  |  | 0.511 | |
|  | A/A | 245 |  |  | |
|  | A/G-G/G | 71 | 0.025[-0.050, 0.101] |  | |
|  | Recessive |  |  | 0.162 | |
|  | A/A-A/G | 308 |  |  | |
|  | G/G | 8 | -0.143[-0.344, 0.057] |  | |
|  | Over-dominant |  |  | 0.216 | |
|  | A/A-G/G | 253 |  |  | |
|  | A/G | 63 | 0.050[-0.029, 0.129] |  | |
|  | log-Additive |  |  | 0.910 | |
|  | 0,1,2 |  | 0.004[-0.061, 0.068] | 0.198 | |
| LDL | Codominant |  |  |  | |
|  | A/A | 245 |  | 0.272 | |
|  | A/G | 63 | 0.054[-0.190, 0.298] |  | |
|  | G/G | 8 | 0.503[-0.117, 1.123] |  | |
|  | Dominant |  |  |  | |
|  | A/A | 245 |  | 0.381 | |
|  | A/G-G/G | 71 | 0.104[-0.129, 0.337] |  | |
|  | Recessive |  |  |  | |
|  | A/A-A/G | 308 |  | 0.120 | |
|  | G/G | 8 | 0.492[-0.126, 1.110] |  | |
|  | Over-dominant |  |  |  | |
|  | A/A-G/G | 253 |  | 0.761 | |
|  | A/G | 63 | 0.038[-0.206, 0.282] |  | |
|  | log-Additive |  |  |  | |
|  | 0,1,2 |  | 0.127[-0.072, 0.326] | 0.211 | |
| TG | Codominant |  |  |  | |
|  | A/A | 245 |  | 0.044 | |
|  | A/G | 63 | -0.136[-0.398, 0.127] |  | |
|  | G/G | 8 | 0.753[0.085, 1.421] |  | |
|  | Dominant |  |  |  | |
|  | A/A | 245 |  | 0.782 | |
|  | A/G-G/G | 71 | -0.036[-0.288, 0.217] |  | |
|  | Recessive |  |  |  | |
|  | A/A-A/G | 308 |  | 0.022 | |
|  | G/G | 8 | 0.781[0.115, 1.447] |  | |
|  | Over-dominant |  |  |  | |
|  | A/A-G/G | 253 |  | 0.236 | |
|  | A/G | 63 | -0.160[-0.423, 0.104] |  | |
|  | log-Additive |  |  |  | |
|  | 0,1,2 |  | 0.055[-0.161, 0.271] | 0.619 | |

CI: Confidence interval; ALT, Alanine aminotransferase; HDL, High-density lipoprotein; LDL, Low-density lipoprotein; NAFLD, Nonalcoholic fatty liver disease; TG, Triglyceride; SNP: Single nucleotide polymorphisms; SOD2: Superoxide Dismutase 2.
